# Word recognition during movement under simulated weather conditions

**DOI:** 10.1371/journal.pone.0326945

**Published:** 2025-07-10

**Authors:** Francisco Rocabado, Natasha Alonso-Bernal, Jon Andoni Duñabeitia

**Affiliations:** 1 Centro de Investigación Nebrija en Cognición (CINC), Department of Education, Universidad Nebrija, Madrid, Spain; 2 AcqVA Aurora Center, UiT The Arctic University of Norway, Tromsø, Norway; University of Minho School of Psychology: Universidade do Minho Escola de Psicologia, PORTUGAL

## Abstract

Processing linguistic materials while driving is essential for ensuring road safety; however, adverse weather conditions can compromise our ability to read at ease. Here we rely on Virtual Reality (VR) to recreate real-world perceptual disfluency, allowing us to investigate the effects of differing weather scenarios—such as sunny and rainy—on word recognition while in movement. Participants navigated a realistic VR driving environment and performed a word identification and naming task, with linguistic materials displayed on road signs encountered along the route. Results showed that high-frequency words were recognized better than low-frequency counterparts, reaffirming the strength of the frequency effect, even under dynamic situations. Additionally, reaction times were slower in rainy compared to sunny conditions, indicating that adverse weather impairs processing speed but not overall recognition accuracy. The negative effect of rain also increased progressively over time, suggesting a potential accumulation of perceptual fatigue or diminished visual adaptation. The lack of a significant interaction between the weather conditions and word frequency suggests that these effects were independent, with no significant interaction between frequency and weather condition. These findings demonstrate the utility of VR as an ecologically valid framework to investigate the complex interplay between environmental context and reading comprehension. In conclusion, the current study provides insights into how varying environmental conditions affect word recognition during movement.

## Introduction

Weather conditions such as rain, fog, snow, and wind not only impact visibility but also influence a driver’s psycho-physiological function, increasing the traffic accident rate by as much as 13% [1]. These adverse conditions can complicate the already challenging task of navigating and responding to road signs and other linguistic cues, which are integral to safe driving. This intersection of environmental and cognitive challenges highlights the importance of understanding how drivers process written material in varying conditions. Besides, the amount of linguistic material in today’s surroundings is vast, and given the fast-paced nature of modern life, it comes as no surprise that much of this material is processed while in motion, whether we are driving or simply riding as passengers. Words on road signs might drift away or move closer to the viewpoint at a millisecond pace depending on the changes in location of the readers induced by motion.

One of the most remarkable aspects of visual word recognition is the human ability to extract meaning even when the visual input is degraded. Laboratory studies have shown that people can recognize words despite considerable alterations to letter forms—such as rotated characters, handwritten input, or digit-for-letter substitutions (the so-called Leet effect; e.g., M4T3R14L)—with only mild costs [2–6]. These effects are readily explained by neurobiological models of word recognition, such as the Local Combination Detector model [7], which posit that letter detectors possess tolerance to distortion. Recent work by Perea et al. [8] demonstrated that subtle spatial distortions—like offsetting letter halves—produce minimal reading costs unless the disruption is extreme, reinforcing the system’s resilience. Together, these findings support the notion that the visual word recognition system is both robust and flexible, adapting dynamically to the level and timing of perceptual constraints.

Although in everyday contexts, the perception of linguistic materials extends beyond their inherent features such as sub-lexical and lexical attributes, including length and frequency, among many others [9,10]. Physical characteristics like font, size, and contrast also play a crucial role [11]. Additionally, environmental factors such as ambient luminance [12], and distractors proximate to the focal target have also been shown to influence reading [13–15]. Recent findings have further highlighted that the effects of these visual features may interact with lexical-level properties. For example, Yap et al. [16] demonstrated that under visually challenging conditions, high-frequency words tend to benefit more than low-frequency words in the fastest responses, while the effect is reduced in slower responses, revealing that visual interference can shift the dynamics of word recognition. Over and above in-lab generated environmental factors, in real-life settings, meteorological conditions further complicate reading, including distortions caused by raindrops, the obscurity of fog or sandstorms, or the accumulation of dust [17]. While much research has focused on the intrinsic properties of to-be-read stimuli, a significant gap remains in understanding the overarching impact of these environmental elements. These factors are omnipresent in daily experiences and are crucial for developing effective reading strategies and understanding reading fluency in ecologically valid environments. Therefore, extending the logic of the laboratory work presented thus far to real-world conditions, meteorological factors such as rain may serve as natural source of visual disruption, affecting word encoding in ways comparable to laboratory manipulations.

Real-world scenarios introduce complexities that are often difficult to incorporate to experiments carried out in laboratory settings. For instance, most experiments are often conducted in controlled and silent two-dimensional conditions, failing to capture the dynamic aspects of language processing in the real world, often governed by motion-induced effects [18,19]. Besides the obvious potential impact of movement that alters the perception of written words in a dynamic manner [20], weather-induced perceptual disfluency conditions also posit a challenge to written words as they act as visual noise (i.e., raindrops or fog) that may fade the print and impair reading [21]. Environmental factors can subtly influence the act of vision and therefore it is expected to distort the act of reading. This notion is supported by classic findings where accident risk increases during rainfall [22,23] but returns to normal levels after the rain has stopped [24]. In fact, recent laboratory findings have demonstrated that readers’ eye-movement and reading patterns are significantly affected by weather conditions [17,25]. Reduced visibility can undermine the effectiveness of visual cues, such as road signs. This reduction in visibility often results from glares that impair a driver’s ability to see clearly, decreasing object contrast and making it more difficult to detect hazards [see 12,26]. Given that reduced visibility can significantly affect visual perception, the interaction between an individual’s movement —such as during driving or walking— and the visual processing of words in the presence or absence of natural noise becomes critical. Furthermore, existing research suggests that some perceptual effects could be even more marked in naturalistic that in-lab contexts [see 12]. Consequently, the current study was set to explore the role of two weather conditions (sunny vs. rainy) as two natural visual fluency modulators in word recognition while reading in a highly realistic simulated motion.

Preceding research has sought to replicate the changing visibility of text encountered in real-world scenarios using techniques like the Progressive Demasking Task (PDT). Developed by Grainger and Segui [27], the PDT involves presenting a word stimulus alongside a masking stimulus, with different cycles in which the exposure time of the word and the mask progressively change; while the mask’s duration gradually decreases, the word’s visibility increases. This process partially simulates the dynamic visibility challenges encountered in real-world reading situations where the reader or the text is in motion. Building upon this foundation, recent studies have taken significant steps towards exploring these real-world effects such as the impact of motion, distance, and environmental visibility in more ecologically valid contexts. Tumas [1] directly addressed how factors like distance and environmental conditions impact perceptual accuracy of text, moving beyond the controlled conditions of PDT to more closely replicate actual reading scenarios encountered daily. Continuing this line, Tejero, Royo and Roca [19] investigated dynamic reading in driving scenarios. Their study focused on how drivers perceived and processed textual information (such as road signs or digital displays) under various environmental and motion conditions. By examining word recognition in these dynamic, real-world contexts, their study provided crucial insights into how movement, distance, and changing visibility interact to affect reading performance in practical situations. Specifically, these studies demonstrate that both distance and environmental instability significantly degrade reading accuracy and speed, highlighting the need for clearer text designs and strategic placement of information to accommodate real-world conditions such as driver movement, varying distances, and fluctuating visibility. Altogether, these studies represent a significant progression from laboratory-based tasks to more ecologically valid research paradigms, allowing for a more comprehensive exploration of how environmental conditions and motion influence visual perception and reading comprehension in highly dynamic everyday settings.

All in all, the present study aims to explore the impact of simulated meteorological conditions on word recognition during movement by relying on a Virtual Reality (VR) environment that aids the recreation of realistic weather scenarios while driving. Participants were immersed in a simulated driving scenario and asked to identify the words that were presented on a series of panels and to read them aloud. Our focus pivots on the dynamic processing of high and low-frequency words, examining how familiar versus less familiar words are processed under varying visual conditions of rain and sunshine. The frequency effect serves as a well-established robust marker of lexical processing [28,29], showing that high-frequency words are recognized more quickly and accurately than low-frequency words. This effect was taken as a key indicator to assess how different environmental conditions may modulate word processing efficiency while in a moving vehicle. In fact, prior studies have demonstrated that under particularly degraded conditions—such as difficult-to-read handwriting—interactions emerge between stimulus format and lexical variables like word frequency. Specifically, word frequency effects tend to be amplified, suggesting the engagement of top-down lexical feedback mechanisms to compensate for weak perceptual input [6,30]. In contrast, when perceptual degradation is mild or moderate, these effects often remain additive, implying that recognition is resolved during early, pre-lexical stages. Therefore, identifying whether frequency and weather effects interact or remain additive can offer valuable insight into the locus of interference caused by meteorological conditions.

Based on Sternberg’s additive factors logic [31], we reasoned that if the effects of weather and frequency are additive, this would suggest that adverse weather conditions primarily affect early perceptual stages, prior to lexical access. In contrast, an interaction would indicate that weather impacts processing at the lexical level, modifying the frequency effect. Thus, our study not only examines whether simulated rain affects word recognition but also aims to clarify which stage of processing is disrupted by environmental interference. We hypothesized that both the perceptual visual noise caused by different weather conditions and the motion itself would influence recognition rates, with frequency effects serving as a determinant marker of the differences induced by the experimental conditions. Thus, this study aimed at extending our understanding of the complex interplay between environmental conditions and dynamic settings, providing insights into the cognitive mechanisms that underpin everyday reading activities. Finally, it contributes to the growing body of research on reading under degraded visual input by testing whether well-established cognitive mechanisms for recognizing noisy words also apply in dynamic, real-world conditions like driving.

## Methods

### Participants

A total of 33 students from Nebrija University participated in the present experiment. Twenty-two participants self-identified as female (M_age_ = 21.41, SD = 3.39) and eleven participants self-identified as male (M_age_ = 25.09, SD = 6.33). All participants had normal or corrected-to-normal vision and hearing, and none reported any form of cognitive dysfunction, assessed with a computerized cognitive battery (CogniFit Inc., San Francisco, US). Participants were recruited between May and July, 2024. Before starting the session, participants were informed about the characteristics of the task and provided written informed consent for data collection. All participants were legally adults (i.e., 18 years or older) at the time of the study; therefore, no parental or guardian consent was required. The study protocol was approved by the Research Ethics Committee at Nebrija University (approval code: UNNE-2022–0017). No deviations from the approved protocol were reported. All data were collected directly by the research team in accordance with applicable ethical guidelines and tool usage terms. No external datasets were used.

### Materials

Four hundred Spanish words of high frequency (200 words) and moderated to low frequency (200 words) were selected as target items. The word stimuli were obtained from the SPALEX database [9], selecting only those with high recognition scores. The properties of the words (i.e., word frequency, orthographic neighborhood and word length) were obtained from EsPal [32]. These 400 items were then divided into two lists of 200 words each, with 100 words of each frequency type in each list. Different weather conditions were associated with each list in a random order across participants. Words were matched for their core characteristics within and across lists (all ps > .10; see Table 1).

**Table 1:** Descriptive statistics for word stimuli by list and frequency type.

Frequency type	List	Word frequency	Orthographic neighborhood	Word length
High-frequency	**1**	5.04 (0.21)	1.77 (0.26)	6.50 (0.50)
	**2**	5.03 (0.18)	1.77 (0.29)	6.43 (0.50)
Low-frequency	**1**	2.89 (0.09)	1.78 (0.29)	6.56 (0.50)
	**2**	2.90 (0.07)	1.79 (0.22)	6.47 (0.50)

Means are reported together with standard deviations (in parentheses). Frequency was measured through Zipf logarithmic scale [see 33]. The orthographic neighborhood, represented by OLD20, reflects the average orthographic distance to the 20 nearest neighboring words for each target word [see 34]. Word length is measured in number of letters.

### Virtual reality setting

**Fig 1:**
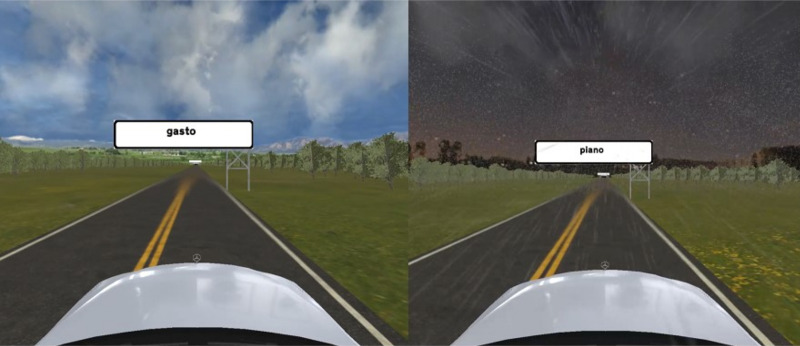
Participant’s view within the experiment. The image on the left shows a sunny weather scenario, while the image on the right shows a rainy weather scenario.

The experimental environment was developed and presented in VR through a head-mounted display (HMD). Experimental stimuli were displayed along high-way road boards on top of the driving lane, under two weather conditions, sunny and rainy. To ensure realism and inmersiveness, the simulated environment included different 3D materials such as trees or bridges. Additionally, ambient sounds were added to the VR environment to enhance the experiment’s realism under both weather conditions. Moreover, the background sky was animated to match both weather scenarios (see Fig 1, S1 File, and S1 File as supporting information).

Python 2.7 and Vizard 6 were used to program and design the VR task [35]. The 3D environments, including all experiment-related content, were presented through the HTC VIVE Pro HMD, with a rendering resolution of 2880x1600 pixels (1440x1600 pixels per eye). The headset’s built-in display offers a 90-Hz refresh rate and a 110° field of view. Notably, participants’ viewpoints remained continuously anchored throughout the VR environment, regardless of any changes in their real-world position.

The experiment was run on a dedicated high-performance desktop PC. This system was equipped with an Intel Core i7-11700F processor running at 2.5 GHz and was cooled using a Corsair Hydro H100x liquid cooling kit. 32GB of RAM and an MSI GeForce 3080 VENTUS 3X PLUS LHR graphics card with 10GB GDDR6X memory. For storage, the PC utilized a 2TB SSD, ensuring fast data access and processing speeds critical for maintaining the real-time performance demands of the VR environment, thus, reducing the probabilities of cybersickness because of delay latency.

### Task and procedure

Participants wore the head-mounted display while seated on a rotating chair to immerse themselves in the described 3D virtual environment, allowing for a complete 360° view from a fixed position as if they were seating in a car. Participants occupied the front travel seat. Each word would be displayed centered on a white traffic board in black Traffic Type W 01 Spain D font, which was selected because it is the standard font used on road signs in Spain. This choice not only ensured that the text reflected the visual characteristics of actual traffic signs, but it also contributed to the ecological validity of the study.

**Fig 2:**
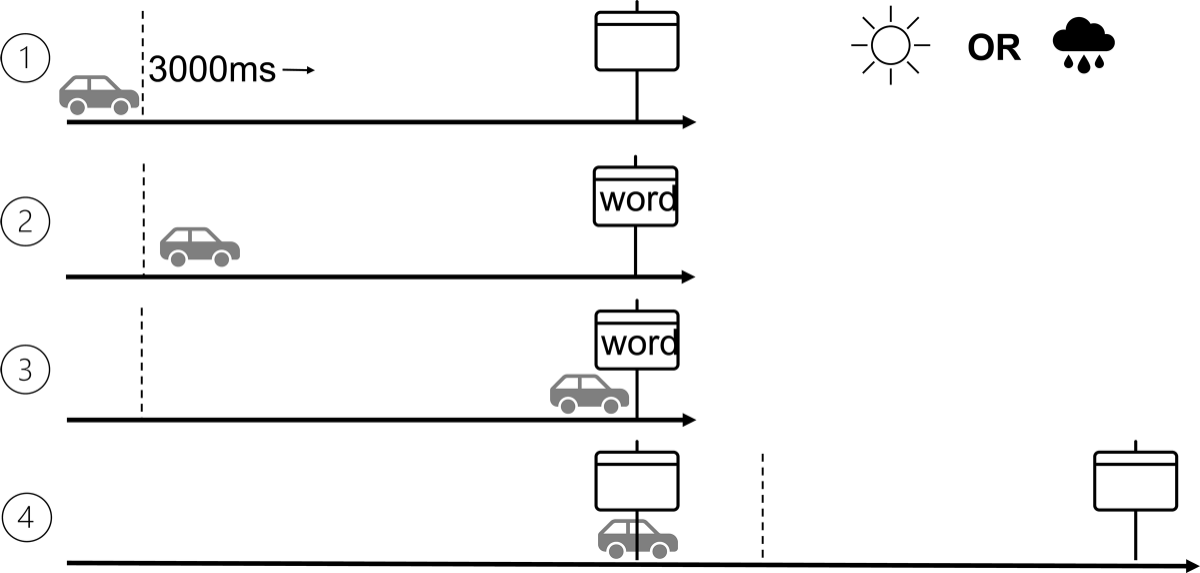
Experimental setup structure of word displays triggered by the VR camera within the simulated scenario. As the car activated the word display by crossing an invisible border, participants were given 3 seconds to respond by pulling a trigger and then name the word. The word remained visible for up to 3 seconds, matching the moment the camera collided with the traffic board.

After placing and calibrating the headset, participants were provided with two controllers, representing two hands in the virtual setting. Subsequently, they were presented with instructions for a naming task on a floating canvas. Once the instructions were read, the car would start moving at a fixed speed of 25 units per second (equivalent to approximately 90 km/h, with one unit corresponding to one meter in virtual space). This speed was selected to reflect typical Spanish intercity driving on two-lane roads, matching the layout of the VR environment. Invisible boundaries were placed on the lane that would interact with the VR camera, triggering both word display at a distance of 75 units and its concealment once the VR camera crossed the traffic board. Participants were instructed to pull the right controller’s trigger as soon as they were able to read each word. In the event of the trigger being pulled, the word would disappear, and participants would have time to read aloud the displayed word. From the moment the word appeared, participants had 3 seconds to respond, which corresponded to the time until matching the boundary under the traffic board (see Fig 2).

## Results

The resulting data files were pre-processed and cleaned using R (R Core Team, 2022) within the RStudio environment [36]. Naming accuracy was automatically assessed with the audio.whisper package [37] that allows audio transcription relying on OpenAI automatic Speech Recognition model [38]. If the transcription matched the expected words, it was marked as correct. Additionally, all audios were manually assessed to double-check for naming accuracy. An outlier cleaning procedure was applied to the correct responses and participants’ reaction times that were below 100 ms and 2.5 SD faster or slower than the mean RT per condition or those associated with timed-out responses were excluded from the latency analysis. This process resulted in 3.33% of the data being excluded.

We conducted mixed-modelling analyses for both accuracy and reaction times in Jamovi [39] using the GAMLj module [40]. For each outcome, we specified a fixed-effects structure that included the two-level factors Frequency (High vs. Low) and Weather Condition (Sunny vs. Rainy), as well as a continuous predictor representing the standardized stimulus presentation order. All two-way interactions among these predictors were included in the model, and the three-way interaction was excluded as it impoverished both models’ fit. For accuracy, a generalized linear mixed-effects model with a binomial distribution and logit link was used. The final model included random intercepts for participants and items, providing the best fit (AIC = 2000.44) without convergence or singularity issues. For reaction times, a linear mixed-effects model was fitted using restricted maximum likelihood (REML). The final model included random intercepts for items and random intercepts and slopes for both Frequency and Weather Condition by participant, showing the best fit (AIC = 1772.21) and explained a substantial proportion of variance (Conditional R^2^ = 0.669), without overfitting. Descriptive statistics for both accuracy and reaction times are presented in Table 2.

**Table 2:** Descriptive analysis of mean reaction times (in milliseconds) and accuracy proportions across frequency type and weather conditions.

Frequency	Weather condition	Reaction times M (SD)	Accuracy M(SD)
**High**	Rainy	1200 (406)	0.99 (0.08)
	Sunny	1073 (366)	0.99 (0.07)
**Low**	Rainy	1410 (479)	0.97 (0.16)
	Sunny	1289 (459)	0.97 (0.16)

Standard deviations are presented in parentheses.

**Fig 3:**
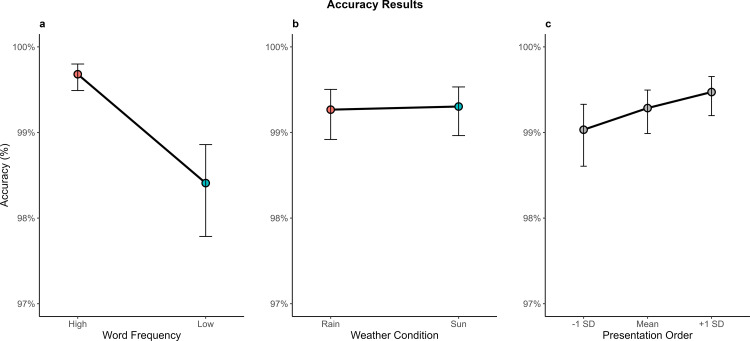
Mean accuracy percentages represented across different experimental conditions. **(a)** Word Frequency, **(b)** Weather Condition, and **(c)** Presentation order. Vertical bars represent 95% confidence intervals.

Accuracy data (n = 13,048 observations) analysis revealed a significant main effect of Frequency, χ²(1) = 57.23, p < .001, with participants responding more accurately to high-frequency words than to low-frequency words. Post hoc comparisons indicated a difference of 2.1 percentage points between the two frequency levels (see Fig 3A). No significant main effect of Weather Condition was found, χ²(1) = 0.07, *p* = .784 (see Fig 3B), Finally, there was a significant main effect of stimulus Presentation Order, χ²(1) = 10.34, p = .001, indicating that accuracy slightly improved over the course of the experiment (see Fig 3C). Estimated marginal means showed an increase from 99% of accuracy (at –1 SD) to 99.5% (at +1 SD), suggesting a modest but reliable trend toward increased accuracy as the task progressed.

As per the interaction, none were statistically significant. The interaction between Frequency and Weather Condition was non-significant, χ²(1) = 0.051, *p* = .821, suggesting that the frequency effect was consistent across weather conditions. Similarly, the interaction between Presentation Order and Weather Condition was not significant, χ²(1) = 1.842, *p* = .175, indicating that the pattern of improved accuracy over time did not differ between sunny and rainy phases. Finally, the interaction between Presentation Order and Frequency was negligible, χ²(1) < 0.001, *p* = .991, indicating that the frequency effect remained stable across the progression of the task.

**Fig 4:**
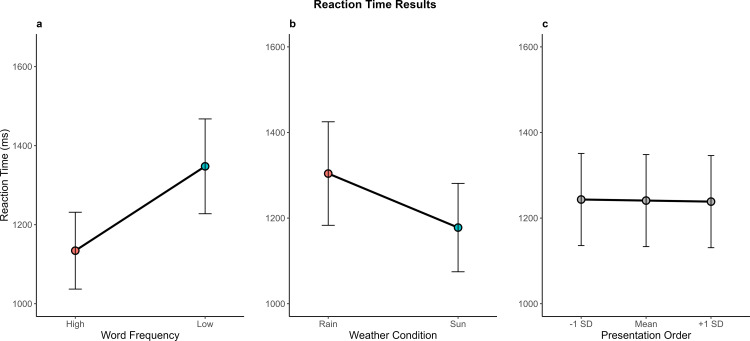
Mean reaction times in milliseconds (ms) represented across different experimental conditions. **(a)** Word Frequency, **(b)** Weather Condition, and **(c)** Presentation order. Vertical bars represent 95% confidence intervals.

Reaction time data (n = 12,620 observations) analysis revealed a significant main effect of Frequency, *F*(1, 63.9) = 120.80, *p* < .001, with participants responding 213 ms faster to high-frequency words than to low-frequency words (see Fig 4a). A significant main effect of Weather Condition was also found, *F*(1, 31.9) = 14.20, *p* < .001, with participants responding 126 ms faster in sunny conditions than in rainy ones (see Fig 4b). The main effect of Presentation Order was not significant, *F*(1, 12,247) = 1.10, *p* = .295 (see Fig 4c).

**Fig 5:**
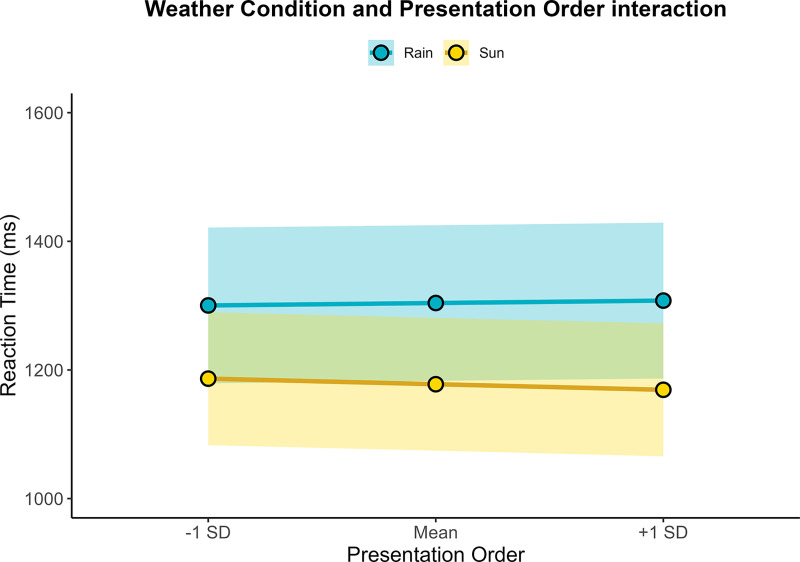
Reaction times interaction between Weather Condition and Presentation Order across three points (–1 SD, Mean, **+ 1 SD).** The rain condition is presented in blue whereas the sun condition in yellow. Shaded areas represent 95% confidence intervals.

Regarding interaction effects, no significant interactions were observed between Frequency and Weather, *F*(1, 12,126.3) = 0.72, *p* = .396, or between Frequency and Presentation Order, *F*(1, 12,235.8) = 0.28, *p* = .594. However, a significant interaction was found between Weather Condition and Presentation Order, *F*(1, 12,245.7) = 7.05, *p* = .008 (see Fig 5). Follow-up simple effects analyses showed that the effect of Weather Condition became stronger as the task progressed. At the beginning of the task (–1 SD), participants were 114 ms faster in sunny than rainy conditions, *t*(33.1) = –3.36, *p* = .002, 95% CI [–183, –45]; this difference increased to 126 ms at the midpoint of the task, *t*(31.9) = –3.77, *p* < .001, 95% CI [–194, –58], and to 139 ms in the final stages of the task (+1 SD), *t*(33.1) = –4.10, *p* < .001, 95% CI [–207, –69.8]. These findings suggest that the negative effect of rain on processing speed becomes more pronounced over time, potentially due to increased perceptual fatigue in degraded visual conditions.

## Discussion

The present study examined how varying weather conditions and word frequency affect cognitive processing during dynamic reading tasks in a virtual driving environment. In psycholinguistic research, tasks that progressively reveal a word or portions of it have been classically used to study the incremental nature of word recognition, allowing insights into how visual information accumulates to reach recognition thresholds. To parallel those studies through a more ecologically valid scenario, we have relied on VR technology. Using controlled and highly immersive VR scenarios, we explored how word recognition unfolds under two levels of environmental visual conditions—sunny and rainy—simulated through immersive VR. This approach provided valuable insights into how environmental factors can influence cognitive tasks, such as reading in motion. By integrating VR and psycholinguistic measures, our approach offers new insights of how environmental factors modulate cognitive processing during tasks that require real-time language comprehension in complex, ecologically valid settings.

Within the present driving simulated scenario participants were prompted to recognize words of high and low frequency displayed on traffic signs under two different weather conditions—sunny and rainy. As expected, and in line with preceding research showing consistent frequency effects during single-word recognition [see 41,42], results showed that high-frequency words were recognized more quickly and slightly more accurately than low-frequency ones under dynamic perceptual conditions. This reinforces the robustness of frequency effects in facilitating word recognition, even under challenging environmental conditions that simulate real-world features. Furthermore, the significant main effect of weather conditions on word recognition, showing that participants took longer to identify words in rainy than in sunny conditions, align well with previous VR studies on reading and word recognition on steady conditions, indicating that adverse weather scenarios can impair visibility and consequently linguistic material processing (Rocabado et al., 2024). Similarly, these findings are consistent with previous studies on driving behaviour, which show that drivers exhibit decreased performance during adverse weather conditions [e.g., 22,23].

The lack of a significant interaction between meteorological conditions and word frequency suggests that these factors affect word recognition independently. This could be tentatively explained by early visual processing stages and mechanisms as delineated in current reading models. Event-Related Potential (ERP) components like P1 and N1 (100–170ms post-stimulus presentation) reflect initial visual processing stages where content and context are integrated [43,44]. Although we did not record neural activity, based on preceding ERP findings we hypothesize that the visual noise caused by rain likely disrupts early stages of processing, affecting all words equally regardless of their frequency. Following Sternberg’s additive factors logic [31], this implies that rain impairs the initial visual encoding of stimuli before lexical access is engaged. This aligns with models of visual word recognition that assume a bottom-up accumulation of orthographic evidence (see [7], Local Combination Detector model), modulated only later by lexical familiarity. Recent findings by Fernández-López et al. [45] further support this interpretation: their results show that distorted forms resembling CAPTCHAs can still activate lexical representations, with frequency modulating this activation only when the visual input is severely degraded. In our study, the perceptual degradation induced by rain may have impaired visual acuity without reaching the threshold necessary to engage top-down lexical compensation, thereby resulting in additive effects between weather and word frequency.

All in all, these results suggest that drivers may be more susceptible to distractions and slower in processing linguistic information during rainy conditions. This highlights the importance of considering the design of road signs and other navigational aids that can improve safety and cognitive performance. For example, adaptive traffic systems that could adjust signage readability based on current weather conditions by altering font size, brightness, or contrast, in response to real-time visibility conditions could be developed. Previous research has highlighted the potential for integrating findings with intelligent transportation systems (ITS) designed to improve traffic efficiency and safety by adapting to real-time environmental conditions [46]. These systems aim to mitigate the negative effects of adverse weather conditions—such as reduced visibility—by assessing how rain and fog impact traffic parameters. Additionally, recent advancements in traffic sign recognition, like Zhang et al. [47] algorithm for recognizing traffic signs in rainy conditions and Redmon et al [48]. You-Only-Look-Once (YOLO) system, demonstrate the progress made in this area. The findings of the present study could further contribute to developing more resilient traffic sign recognition systems, which would account for various weather conditions and their effects on reading comprehension. Building on this, the present findings could inform the development of adaptive signage systems that adjust visual properties in real-time, enhancing readability across varying weather conditions.

Beyond the main effects of frequency and weather, we also observed time-on-task effects that influenced performance across the experiment. Accuracy improved slightly but significantly over time, suggesting a modest learning or adaptation trend as participants became more familiar with the task. While stimulus order did not significantly affect reaction times overall, a significant interaction emerged between weather condition and stimulus order. Specifically, the negative impact of rainy conditions on response times became increasingly pronounced as the task progressed, indicating that prolonged exposure to visual degradation may result in accumulated perceptual fatigue or reduced adaptation efficiency. Importantly, frequency effects remained stable over time, suggesting that lexical familiarity consistently facilitates word recognition regardless of task progression. These findings underscore the importance of accounting for temporal dynamics when evaluating cognitive performance under real-world perceptual challenges.

In conclusion, while the application of VR in psychological research is still emerging, with ongoing efforts to replicate classic cognitive effects [21,49–51], this investigation opens up new avenues for understanding how environmental factors like weather influence reading comprehension in dynamic, real-world scenarios like driving. By utilizing VR technology, we have been able to simulate and study complex interactions in a controlled yet realistic manner, advancing our understanding of human cognitive performance in real-life situational contexts —reading in movement. This research not only prompts the development of better cognitive and reading models that account for environmental variables but also informs practical applications that enhance safety and performance in everyday activities like driving.

Building on these findings, future research could examine whether more extreme weather conditions such as fog or snow elicit interactions between perceptual degradation and word frequency, as such scenarios may exceed the tolerance threshold of early visual processing and require compensatory lexical mechanisms. Additionally, it would be valuable to explore whether individuals familiarity with such weather conditions modulates these effects. For example, readers accustomed to environments with frequent fog, snow, or heavy rain may develop perceptual strategies or tolerance that reduce the cognitive cost of visual degradation, compared to those less experienced with such conditions.

## Supporting information

S1 File Sunny weather task. Video sample of the virtual reality task under sunny weather condition.
(MP4)

S2 File Rainy weather task. Video sample of the virtual reality task under rainy weather condition.
(MP4)

S3 File Inclusivity in global research. (PDF)
